# Plasma Lipolysis and Changes in Plasma and Cerebrospinal Fluid Signaling Lipids Reveal Abnormal Lipid Metabolism in Chronic Migraine

**DOI:** 10.3389/fnmol.2021.691733

**Published:** 2021-08-31

**Authors:** Katherine Castor, Jessica Dawlaty, Xianghong Arakaki, Noah Gross, Yohannes W. Woldeamanuel, Michael G. Harrington, Robert P. Cowan, Alfred N. Fonteh

**Affiliations:** ^1^Department of Neurosciences, Huntington Medical Research Institutes, Pasadena, CA, United States; ^2^Department of Neurology, Keck School of Medicine, University of Southern California, Los Angeles, CA, United States; ^3^Pain Center, Department of Neurology, Stanford University, Stanford, CA, United States; ^4^Zilkha Neurogenetic Institute, University of Southern California, Los Angeles, CA, United States

**Keywords:** chronic migraine, lipid signaling, lipolysis, lipases, metabolic syndrome, platelet-activating factor, phospholipase A2, insulin resistance

## Abstract

**Background:**

Lipids are a primary storage form of energy and the source of inflammatory and pain signaling molecules, yet knowledge of their importance in chronic migraine (CM) pathology is incomplete. We aim to determine if plasma and cerebrospinal fluid (CSF) lipid metabolism are associated with CM pathology.

**Methods:**

We obtained plasma and CSF from healthy controls (CT, *n* = 10) or CM subjects (*n* = 15) diagnosed using the International Headache Society criteria. We measured unesterified fatty acid (UFA) and esterified fatty acids (EFAs) using gas chromatography-mass spectrometry. Glycerophospholipids (GP) and sphingolipid (SP) levels were determined using LC-MS/MS, and phospholipase A_2_ (PLA_2_) activity was determined using fluorescent substrates.

**Results:**

Unesterified fatty acid levels were significantly higher in CM plasma but not in CSF. Unesterified levels of five saturated fatty acids (SAFAs), eight monounsaturated fatty acids (MUFAs), five ω-3 polyunsaturated fatty acids (PUFAs), and five ω-6 PUFAs are higher in CM plasma. Esterified levels of three SAFAs, eight MUFAs, five ω-3 PUFAs, and three ω-6 PUFAs, are higher in CM plasma. The ratios C20:4n-6/homo-γ-C20:3n-6 representative of delta-5-desaturases (D5D) and the elongase ratio are lower in esterified and unesterified CM plasma, respectively. In the CSF, the esterified D5D index is lower in CM. While PLA_2_ activity was similar, the plasma UFA to EFA ratio is higher in CM. Of all plasma GP/SPs detected, only ceramide levels are lower (*p* = 0.0003) in CM (0.26 ± 0.07%) compared to CT (0.48 ± 0.06%). The GP/SP proportion of platelet-activating factor (PAF) is significantly lower in CM CSF.

**Conclusions:**

Plasma and CSF lipid changes are consistent with abnormal lipid metabolism in CM. Since plasma UFAs correspond to diet or adipose tissue levels, higher plasma fatty acids and UFA/EFA ratios suggest enhanced adipose lipolysis in CM. Differences in plasma and CSF desaturases and elongases suggest altered lipid metabolism in CM. A lower plasma ceramide level suggests reduced *de novo* synthesis or reduced sphingomyelin hydrolysis. Changes in CSF PAF suggest differences in brain lipid signaling pathways in CM. Together, this pilot study shows lipid metabolic abnormality in CM corresponding to altered energy homeostasis. We propose that controlling plasma lipolysis, desaturases, elongases, and lipid signaling pathways may relieve CM symptoms.

## Introduction

Chronic migraine (CM) is common (Lipton et al., 2016), disabling, challenging to treat (May and Schulte, 2016; Sun-Edelstein and Rapoport, 2016), and presently the focus of promising receptor-mediated signaling pathways studies (Cady et al., 2014). Due to an incomplete understanding of causative mechanisms, it is essential to consider alternative approaches to treat such a disabling condition. Lipid disturbances in episodic migraine include endocannabinoids (Cupini et al., 2008; Akerman et al., 2013) and lipases (Fonteh et al., 2011; Haghdoost et al., 2016), suggest that their involvement in CM may offer new treatment options.

Lipids are sources of bioactive molecules associated with pain, inflammation, vascular activity, neuroplasticity, ion channel, and receptor functions (Mead and Dhopeshwarkar, 1971; Rouser et al., 1972; Piomelli and Sasso, 2014; Schroeder and Brunet, 2015). These properties are associated with headache pathophysiology, but the relationship of different fatty acids, glycerophospholipid (GP), and sphingolipid (SP) profiles with CM are not obvious. Furthermore, fatty acyls that are the building blocks of more complex GPs, SPs, are also modifiers of signaling proteins (Boutaud et al., 2005; Liebisch et al., 2020). Thus, measurement of saturated fatty acid (SAFA), monounsaturated fatty acid (MUFA), or polyunsaturated fatty acid (PUFA) when unesterified fatty acid (UFA) or as esterified fatty acid (EFA) presents an opportunity to understand CM pathophysiology better.

The most studied signaling lipids derived from PUFAs are collectively known as eicosanoids and are synthesized through enzyme activities (Rubin and Laposata, 1992). Eicosanoids from ω-6 (omega-6) fatty acids are generally inflammatory, while ω-3 (omega-3) derived species are anti-inflammatory and, in some cases, resolve inflammation (Arita et al., 2005; Berthelot et al., 2015; Devassy et al., 2016). Eicosanoids are also linked to pain and induce headaches in animal and human migraine models (Wienecke et al., 2010; Antonova et al., 2012; Myren et al., 2012; Kheirollahi et al., 2015). Widely used non-steroidal anti-inflammatory drugs (NSAIDs) reduce the formation of one class of eicosanoids (prostaglandins) via prostaglandin synthase (cyclooxygenase) inhibition (Vardi et al., 1976). However, knowledge of upstream fatty acid precursors of eicosanoids in headache disorders is limited. We propose that examination of upstream lipid metabolism may offer alternative approaches for preventing migraines.

Genetics, epigenetics, and environmental/dietary factors may influence the upstream PUFA precursors (Huang et al., 2015; Liu et al., 2015; Lemas et al., 2016). PUFA precursors are essential nutrients that are not endogenously synthesized and are only obtained from the diet. Various desaturation and elongation enzymes compete to convert PUFA precursors to longer chain ω-6 and ω-3 PUFAs. As the rate-limiting steps, delta-5-desaturase (D5D) and delta-6-desaturase (D6D) are essential in regulating PUFA composition in cells and tissues (Alarcon et al., 2016; Lemas et al., 2016; Monk et al., 2016). Other desaturases, including delta-9-desaturase (D9D) or stearoyl-CoA desaturase (SCD), regulate the energy requirement of cells and interact with insulin to influence glucose homeostasis and metabolic syndromes (Kawashima et al., 2009; de Moura et al., 2016).

Another upstream process that may regulate brain signaling is the hydrolysis of complex lipids mediated by phospholipases (Dennis et al., 2011; Bollag, 2016; Chap, 2016). Phospholipase A_2_ (PLA_2_) is usually closely associated with regulating UFA levels and eicosanoid formation initiation. Activation of calcium-dependent PLA_2_ is associated with neuropathic pain, inflammation, and several neurovascular diseases (Lucas et al., 2005; Svensson et al., 2005; Yaksh et al., 2006). PLA_2_ is also associated with autocrine functions, including immune response, insulin sensitivity, likely via receptor-mediated activation of receptor-mediated signaling pathways (Bao et al., 2006; Ibeas et al., 2009; Shridas et al., 2014; Kuefner et al., 2017, 2019). Neurotransmitter (serotonin and glutamate) and neuroinflammatory peptide (CGRP, substance P, bradykinin) receptor signaling associate with migraine pathophysiology and also involve phospholipase pathways (Kim et al., 1997; Wei et al., 2003; Locker et al., 2006; de Vries et al., 2020). Thus, any plasma or cerebrospinal fluid (CSF) change in PLA_2_ activity in CM will provide insight into pain and inflammatory signaling pathways.

In addition to PLA_2_ that hydrolyzes phospholipids, the sequential hydrolysis of triacylglycerol (TAG) in lipid droplets releases fatty acids. The three main enzymes that hydrolyze TAG are adipose triglyceride lipase (ATGL), hormone-sensitive lipase (HSL), and monoacylglycerol lipase (MAGL). ATGL hydrolyzes TAG to release a fatty acid and diacylglycerol (DAG). HSL hydrolyzes DAG to release a fatty acid and monoacylglycerol (MAG) that MAGL subsequently hydrolyzes to release a fatty acid and glycerol (Coleman and Mashek, 2011; Quiroga and Lehner, 2018). These lipolytic activities generate signaling molecules that regulate metabolism in many tissues (Choi et al., 2010; Rogne and Tasken, 2014; Herzer et al., 2015). For example, in adipocytes, CGRP is involved in mobilizing lipids (Walker et al., 2014). Thus, lipolysis is vital in energy homeostasis as well as generating signaling lipids.

With the studies above linking lipid metabolism with pain, inflammation, and receptor-mediated signaling pathways, we propose that lipid metabolism is different in CM than in healthy non-headache controls (CT). Thus, we aim to quantify fatty acids, glycerophospholipids, and sphingolipids in plasma and CSF to determine any lipid interaction with CM pathophysiology.

## Materials and Methods

### Recruitment and Clinical Classification

We recruited study participants for an IRB-approved cross-sectional study at the Stanford University Headache and Facial clinic. All participants gave written informed consent before enrollment and were recruited as non-headache controls (CT) or those with CM based on the International Classification for Headache Disorders, second edition (ICHD-3 beta) criteria (Olesen and Third International Headache Classification Committee of the International Headache, 2011). Exclusion criteria included the use of opioids and other non-migraine-related neurological disorders. Control participants were healthy individuals (>18 years) with no history of headaches. In addition, participants were not fasting when we collected CSF and plasma for both CT and CM.

### Cerebrospinal Fluid Collection

We obtained CSF by lumbar puncture between 8.00 am and 4.00 pm, centrifuged to remove any cellular debris, and then fractionated into 1 mL aliquots before storage at −80°C.

### Plasma Collection

We collected whole blood by venous puncture into EDTA-treated (lavender tops) anticoagulant tubes. Removal of blood cells/platelet depletion to obtain a plasma layer was achieved by centrifugation for 15 min at 2,000 × *g* at 4°C. The supernatant fluid (plasma) was immediately transferred in 0.5 ml aliquots into a clean polypropylene tube. The plasma aliquots were stored at −80°C until required for lipid and enzyme analyses.

### Protein Assay

Cerebrospinal fluid was diluted (2×) with TBS, and protein levels were determined using a Quant-iT fluorescent assay (Invitrogen/Molecular Probes, Eugene, OR, United States) with bovine serum albumin (0–500 ng/ml) as a standard.

### Lipid Extraction and Derivatization

Lipids in 25 μl plasma or 1 mL CSF were extracted using a modified Bligh and Dyer method (Bligh and Dyer, 1959). Deuterated fatty acid standards (100 ng each) were added to monitor recovery and GC/MS quantification of fatty acids. We performed derivatization by adding 50 μL each of a solution of 10% *N,N*-diisopropylethylamine in acetonitrile, and 5% 2,3,4,5,6-pentafluorobenzyl bromide in acetonitrile to the dried lipid extracts, followed by heating to 60°C for 30 min. The solution was dried under a nitrogen stream and then taken up to 100 μL of dodecane for GC/MS analysis.

### Measurement of UFA

To 25 μl plasma in 975 μl Tris-buffered saline or to 1 mL CSF, we added 3 drops of 0.9% formic acid to acidify the solutions before adding 100 ng of each deuterated internal standard in the presence of antioxidant (0.25 mg/mL BHT in methanol). Lipids were extracted and derivatized as described above. UFA levels in CSF were quantified using stable isotope dilution GC/MS as previously described (Fonteh et al., 2014).

### Measurement of EFA

For EFAs, 100 ng deuterated fatty acid standards were added to 10% of lipids extracted from 25 ul plasma or 1 mL CSF. The mixture was hydrolyzed using 0.5 M HCl in acetonitrile (9:1 v/v) at 100°C for 30 min (Aveldano and Horrocks, 1983). Extracted fatty acids containing UFA and EFA were derivatized as described above and quantified using stable isotope GC/MS.

### LC-MS/MS of Glycerophospholipids and Sphingolipids

Glycerophospholipids and SPs in plasma or CSF extracts were separated using a hydrophilic interaction liquid chromatography (HILIC) as previously described (Fonteh et al., 2013). GPs and SPs were eluted from the HILIC column and directly infused to a triple quadrupole mass spectrometer (TSQ Classic, Thermo Fisher Scientific, San Jose, CA, United States). The instrument was operated in the positive mode and scan events for different GPs [phosphatidylcholine (PC), platelet-activating factor (PAF), lyso platelet-activating factor (LPAF), and sphingomyelin (SM) precursor ion scanning *m/z* 184, lysophosphatidylcholine (LPC) precursor ion of 104, ceramide (Cer), precursor ion of 264, and dihydroceramide (dhCer)] with precursor ion scan of 266). The internal standard (PC 11:0/11:0) peak was acquired using selected reaction monitoring (SRM) of *m/z* 595 (precursor ion) to 184 (product ion) (Fonteh et al., 2013).

### Phospholipase A_2_ Activity

Plasma or CSF equivalent of 10 μg total protein was incubated with a fluorescent lipid cocktail, and PLA_2_ activity was kinetically monitored over 1 h (Fonteh et al., 2013). PLA_2_ specific activity (RFU/μg/min) was calculated at the linear portion of the activity profile.

### Fatty Acid Indices

For data reduction, we calculated fatty acid indices that represent desaturation, elongation, the number of carbon, unsaturation, and peroxidation. Desaturase and elongase that measure the desaturation and elongation levels of fatty acids, respectively, were determined by calculating the ratios (desaturase or elongase indices) of products to precursors; D9D = C18:1/C18:0, C16:1/C16:0, or C24:1/C24:0, elongase = (C16:0/C14:0 + C18:0/C16:0 + C20:0/C18:0 + C22:0/C20:0 + C24:0/C22:0)/6, D6D = C18:3n-6/C18:2n-6, D5D = C20:4n-6/homo-γ-C20:3n-6, and D4D = C22:6n-3/C22:5n-3. Fatty acid chain length Chain length index (CLI) = Σ FA × carbon number/total FA concentration (Mocking et al., 2012). The number of double bonds per fatty acid = Unsaturation index (UI) = Σ 1 × Monoenoics + 2 × Dienoics + 3…./Total FA concentration (Mocking et al., 2012). Peroxidation susceptibility = Peroxidation index (PI) = Σ 0.025 × Monoenoics + 1 × Dienoics + 3 × trienoics + 4 × tetraenoics + 6 × pentaenoics + 8 × hexaenoic/Total FA concentration (Mocking et al., 2012).

### Statistical Methods

Plasma and CSF UFA and EFA were normalized to levels from 1 mL (ng/mL) and as the percentage of total UFA or total EFA > C14:0. Mann–Whitney *U* tests were performed to determine significant differences in fatty acid levels in CT compared with CM. One-way ANOVA on ranks (Kruskal–Wallis test) and correction for multiple comparisons with statistical hypothesis testing using Dunn’s method were performed to determine within-group differences of lipid molecular species. We used the Multiple Mann–Whitney test that compares ranks with multiple comparisons adjustment for False Discovery Rate (FDR) using the Two-Stage step-up method of Benjamini, Krieger, Yekutieli. For plasma fatty acids, data normalization was performed using MetaboAnalyst software by first converting Excel sheet data to tab-delimited text (.txt) before importing the text to the MetaboAnalyst Statistical Analysis platform (Chong and Xia, 2020). Fatty acid data normalization and scaling used globalized logarithm transformation (glog) and mean-centering to obtain a Gaussian distribution and compare fatty acid levels over several orders of magnitude in plasma. Hierarchical clustering data presented in the form of a heatmap used Euclidean for distance measure and Ward for the clustering algorithm. All analyses were performed using GraphPad Prism software v 9. (La Jolla, CA, United States) or MetaboAnalyst. Since this is an exploratory pilot study, sample size, study power, and effect size were not computed *a priori*. Lipid data were considered significant if *p* < 0.05 after adjustment for multiple comparisons. We present all lipid measures as the mean ± SEM and the 95% CI.

## Results

### Clinical Data

We collected CSF and plasma from CT (*n* = 10) and CM (*n* = 15) participants at the peak of a typical headache for this study. Table 1 shows the demographic distribution and clinical classification of each study group. Age, the proportion of females, and BMI were similar for CT and CM patients. The CT group did not take any medication, but CM patients took both migraine prophylactic and rescue drugs; the most commonly used prescription medications in CM were NSAIDs in 67% of patients (Table 1). The duration of CM ranged from 1 to 36 years (mean ± SD = 11.4 ± 9.2 years), and 10 out of 15 reported comorbid migraine conditions in the CM group.

**TABLE 1:** Demographic data and clinical classification.

Parameter	CT (*n* = 10)	CM (*n* = 15)	*p* value^1^
Age (mean ± SD, 95% CI)	35.2 ± 14.0 (25.2–45.2)	39.7 ± 15.3 (31.2–48.1)	0.4361
Female (%)	8/10 (80)	14/15 (93)	0.5385^2^
BMI (mean ± SD, 95% CI)	26.1 ± 4.1 (22.3–29.8)	26.5 ± 7.3 (22.3–30.7)	0.6888
Headache duration (years)	0	23.8 ± 15.3 (15–32.6)	N/A
CM duration (years)	N/A	11.4 ± 9.2 (6.1–16.7)	N/A
CM headache severity (out of a 0–10 scale)	N/A	7 ± 1 (6–8)	N/A
CM headache frequency (# of headache days/month)	N/A	28 ± 5 (25–30)	N/A
% CM using NSAIDs	0	67%	N/A

*^1^*p* values were obtained using the Mann–Whitney *t*-test.*

*^2^Fisher’s exact test.*

*N/A, not applicable.*

### Plasma and CSF Fatty Acids

**FIGURE 1:**
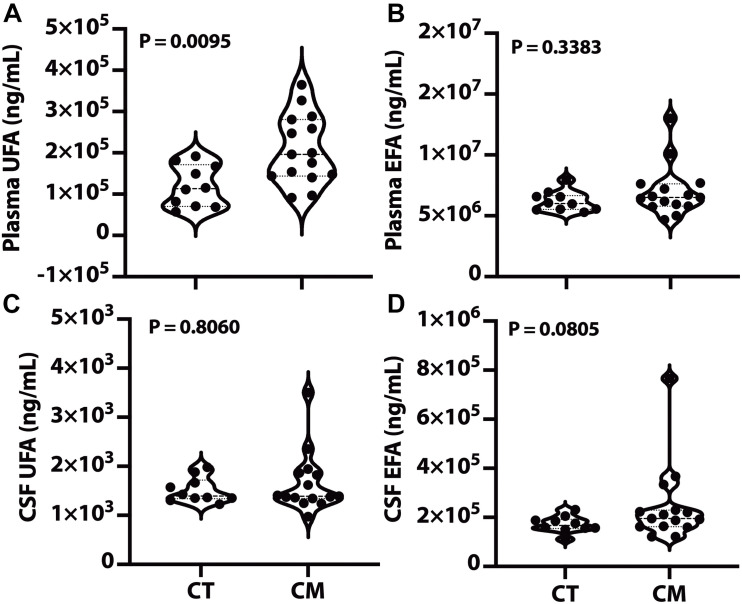
Plasma and CSF unesterified (UFA) and esterified fatty acids (EFA). Lipids were extracted from plasma/CSF, and the levels of the sum of all unesterified or esterified fatty acids > C14:0 were quantified for CT and CM. The violin plots show plasma UFA **(A)**, plasma EFA **(B)**, CSF UFA **(C)**, and CSF EFA **(D)**. In the violin plots, the lower dotted line is the first quartile, the middle line is the median, and the top dotted line is the third quartile. The *p* values were determined using a Mann–Whitney *U* test.

We quantified UFA and EFA levels in plasma and CSF to determine if changes in lipid metabolism are associated with CM pathology. UFA levels were significantly higher in CM plasma than CT (Figure 1A), while plasma EFA levels were not significantly different in CT versus CM. We did not measure any significant difference in UFA and EFA in CSF from CM than CT (Figures 1C,D, respectively).

### Unesterified and Esterified Fatty Acids

**FIGURE 2:**
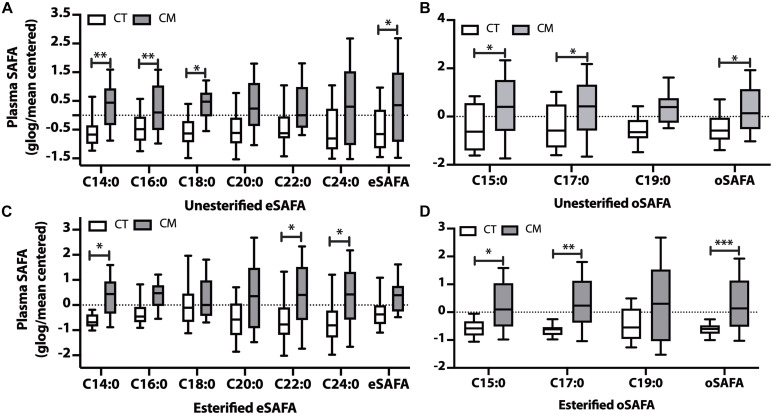
Saturated fatty acid (SAFA) changes in plasma of CT and CM. Box and Whisker plots of unesterified even chain SAFAs (eSAFA) **(A)**, unesterified odd chain SAFAs (oSAFAs) **(B)**, esterified eSAFAs **(C)**, and esterified oSAFAs **(D)**. Fatty acid levels (ng/mL) were normalized using globalized logarithmic transformation and mean-centered. Unpaired multiple *t*-tests with correction for multiple comparisons (False Discovery Rate, FDR) using the two-state step-up method (Benjamini, Krieger, and Yekutieli). ^∗^ denote adjusted *p* (*q*) < 0.05, ^∗∗^ *p* < 0.01, ^∗∗∗^ *p* < 0.005.

**FIGURE 3:**
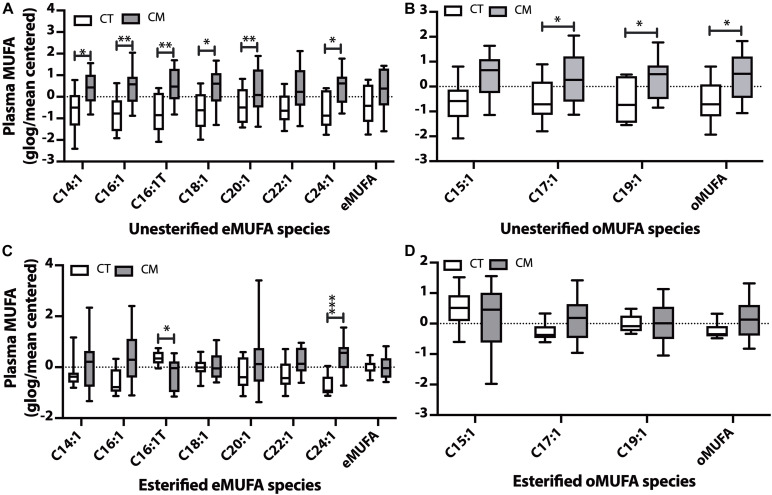
Monounsaturated fatty acid (MUFA) changes in plasma of CT versus CM. We quantified MUFAs in plasma and plotted the normalized (glog/mean-centered) levels for unesterified even chain MUFAs (eMUFA) **(A)**, unesterified odd chain MUFAs (oMUFAs) **(B)**, esterified eMUFAs **(C)**, and esterified oMUFAs **(D)**. Multiple unpaired *t*-tests with correction for multiple comparisons (False Discovery Rate, FDR) using the two-state step-up method (Benjamini, Krieger, and Yekutieli) were used to compare MUFA levels in CT versus CM. One (^∗^), two (^∗∗^), or three asterisks (^∗∗∗^) denote adjusted *p* (*q*) < 0.05, *q* < 0.01, and *q* < 0.005, respectively.

**FIGURE 4:**
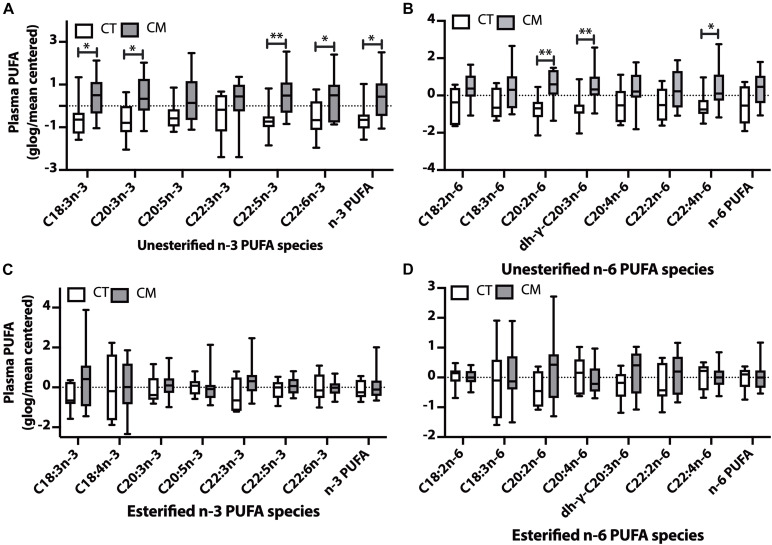
Polyunsaturated fatty acid (PUFA) changes in plasma of CT versus CM. Box and Whisker plots of unesterified n-3 PUFAs **(A)**, unesterified n-6 PUFAs **(B)**, esterified n-3 PUFAs **(C)**, and esterified n-6 PUFAs **(D)**. PUFA levels (ng/mL) were normalized using globalized logarithmic transformation and mean-centered. Unpaired multiple *t*-tests with correction for multiple comparisons (False Discovery Rate, FDR) used the two-state step-up method of Benjamini, Krieger, and Yekutieli. ^∗^ denote adjusted *p* (*q*) < 0.05, ^∗∗^ *p* < 0.01.

To determine if specific groups of fatty acids (SAFA, MUFA, and PUFA) changed in CT compared with CM, we quantified unesterified and esterified SAFAs, MUFAs, and PUFAs in plasma and CSF. For Figures 2–4, the fatty acid levels are normalized (globalized log scale (glog) and mean-centered). The unnormalized data are presented as Supplementary Tables for plasma UFAs (Supplementary Table 1), plasma EFAs (Supplementary Table 2), CSF UFAs (Supplementary Table 3), and CSF EFAs (Supplementary Table 4).

### Plasma Saturated Fatty Acids (SAFAs)

Levels of 3 unesterified even chain saturated fatty acids (eSAFA) (C14:0, C16:0, and C18:0), and the sum of unesterified eSAFA was higher in CM than in CT plasma (Figure 2A). Figure 2B also shows that unesterified levels of two odd chain saturated fatty acids (oSAFAs include C15:0, C17:0) and the sum of oSAFA are higher in CM plasma than in CT. We next examined EFA levels and found an increase in C14:0, C22:0, and C24:0 (Figure 2C), and C15:0, C17:0, and oSAFA in the plasma of CM compared with CT (Figure 2D). These data suggest that levels of some EFAs may change in CM even when total EFA levels do not significantly change.

### CSF SAFAs

Although slightly higher, the levels of unesterified and esterified SAFAs are not significantly altered in CM compared with CT (Supplementary Table 3).

### Plasma Monounsaturated Fatty Acids (MUFAs)

Five unesterified MUFAs (C14:1n-5, C16:1n-7, *trans* C16:1 (C16:1Tn-7), C18:1n-9, C20:1n-9, C24:1n-9, Figure 3A) and C17:1n-9, C19:1n-9, and the sum of oMUFA levels are higher in CM than in CT (Figure 3B). Even though plasma EFAs did not change in CM, we found a significant decrease in C16:1Tn-7 and an increase in C24:1n-9 in CM compared with CT (Figure 3C). There was no significant change in esterified oMUFA levels (Figure 3D).

### CSF MUFAs

Of all the UFAs detected in CSF (Supplementary Table 3), only the levels of C16:1n-7 was significantly higher in CM (1.8 ± 0.5, mean ± SEM, ng/mL, 95% CI = 0.7–3.0) than in CT (1.0 ± 0.1 mean ± SEM, ng/mL, 95% CI = 0.7–1.2, ROC AUC = 0.75 ± 0.1, *p* = 0.0375). Esterified C16:1 levels were also higher in CSF of CM (449.8 ± 92.0 mean ± SEM, ng/mL, 95% CI = 252.4–647.2) than in CT (225.6 ± 22.7 mean ± SEM, ng/mL, 95% CI = 174.3–276.9, ROC AUC = 0.84 ± 0.08, *p* = 0.0037) while the levels of other EFAs were not altered (Supplementary Table 4).

### Plasma Polyunsaturated Fatty Acids (PUFAs)

Plasma unesterified levels of 4n-3 PUFAs (C18:3n-3, C20:3n-3, C22:5n-3, C22:6n-3) and the sum of n-3 PUFAs were higher in CM than CT (4A). Plasma unesterified levels of 3 n-6 PUFAs (C20:2n-6, dihomo-g-C20:3n-6, and C22:4n-6) were higher in CM than CT (4B). There were no significant changes in the levels of esterified n-3 PUFAs (Figure 4C) and esterified n-6 PUFAs (Figure 4D) between CM and CT.

### CSF PUFAs

None of the unesterified n-3 PUFAs detected in CSF were different between groups, although their levels were generally lower in CM (Supplementary Table 3), and only the percent of C18:3n-3 is higher in CM. CSF esterified n-6 PUFA fatty acids were similar in CT and CT (Supplementary Table 3).

### UFA to EFA Ratio in Plasma and CSF

To determine if there is higher hydrolysis or an abnormal buildup of fatty acids compared with EFA storage sites in plasma or CSF, we calculated the ratio of unesterified to the EFA. Plasma UFA to EFA ratio in CT (4.0 ± 1.0 mean ± SEM, 95% CI = 2.9–5.2) is significantly lower than in CM (6.3 ± 2.5 mean ± SEM, 95% CI = 4.9–7.8, *p* = 0.0475). CSF UFA to EFA in CT (0.8 ± 0.2 mean ± SEM, 95% CI = 0.7–0.9) is significantly lower than plasma (*p* = 0.0004) but does not significantly differ from CM (0.7 ± 0.3 mean ± SEM, 95% CI = 0.6–0.9, *p* = 0.3472). These data show a higher turnover of plasma fatty acids than CSF and an increase in their hydrolysis in CM plasma but not in CSF.

### Fatty Acid Indices

To determine fatty acid metabolism in plasma or CSF, we calculated total desaturase indices for CT and CM in plasma and CSF. We also examined the elongase indices to determine if there is a change in the fatty acid chain length.

### Desaturase Indices in Plasma and CSF

**FIGURE 5:**
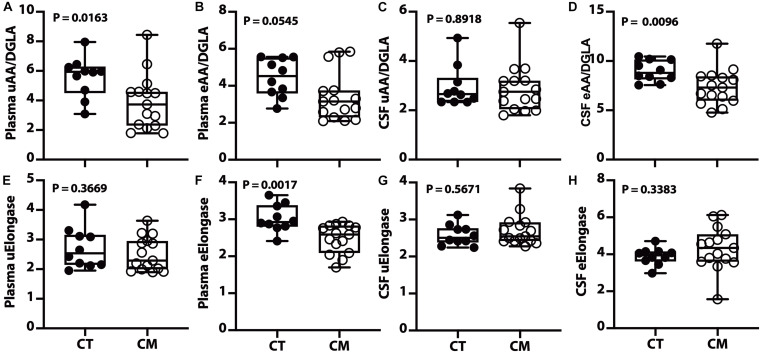
Desaturase and elongase changes in plasma and CSF – Box and Whisker plots of plasma unesterified AA/DGLA **(A)**, plasma esterified AA/DGLA **(B)**, CSF unesterified AA/DGLA **(C)**, and CSF esterified AA/DGLA **(D)**. Violin plots of plasma unesterified elongase index [uElongase, **(E)**], plasma esterified elongase index [eElongase, **(F)**], CSF unesterified uElongase **(G)**, and CSF eElongase **(H)**. The *p* values were determined using a Mann–Whitney *U* test.

Of the four desaturase indices (D4D, D5D, D6D, and D9D) measured in plasma and CSF, only the D5D (AA/DGLA) ratio changed in CM. The plasma unesterified D5D index is lower in CM than CT (Figure 5A). A decreasing trend for plasma esterified AA/DGLA ratio (Figure 5B) is not significant, probably because of 3 outliers in the CM samples. The unesterified CSF D5D is not significantly different (Figure 5C). In contrast, CSF esterified D5D index is significantly lower in CM than in CT (Figure 5D).

### Elongase Indices in Plasma and CSF

We calculated the elongase index in plasma and found no difference between groups for UFAs (Figure 5E). However, there is a significant decrease in plasma esterified elongase index in CM compared to CT (Figure 5F). On the other hand, there is no difference in unesterified (Figure 5G) and esterified (Figure 5H) elongase indices in the CSF.

### Glycerophospholipids (GPs) and Sphingolipids (SPs)

The metabolism of GPs and SPs generate signaling lipids that may impact CM pathology. Therefore, we quantified the principal GP and SP classes in plasma and CSF from CT and CM participants.

### Plasma GPs and SPs

**FIGURE 6:**
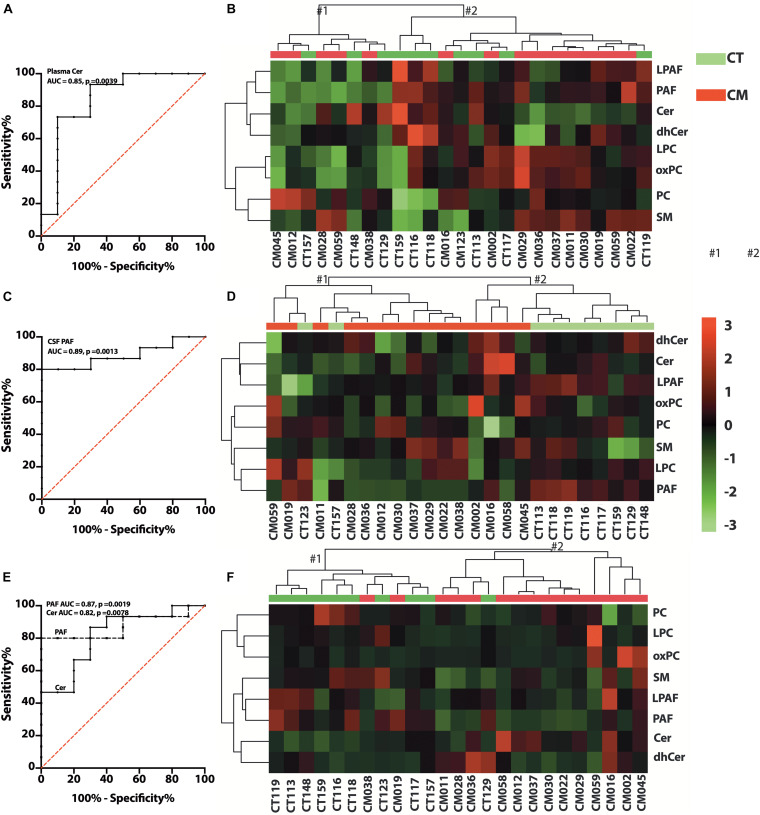
Glycerophospholipid (GP) and SP changes in CT versus CM. **(A)** ROC curve of plasma Cer. **(B)** Heat map of the hierarchical clustering of plasma GPs (LPAF, PAF, LPC, and PC) and SPs (SM, Cer, and dhCer). We used Euclidean for distance measure and Ward for the clustering algorithm for the heatmap. **(C)** ROC curve of CSF PAF. **(D)** Heat map of the hierarchical cluster of CSF GPs and SPs. The distance measure and clustering algorithm for the heatmap used Euclidean for Ward, respectively. **(E)** ROC curve of CSF/plasma of Cer and PAF. **(F)** Heat map of the hierarchical clustering of CSF/plasma ratio of GPs and SPs using Euclidean for distance measure and Ward for the clustering algorithm.

We quantified choline-containing lipid classes in plasma and determined the difference in CT compared with CM participants. The proportion of PC, LPC, LPAF, and PAF in plasma was not significantly different in CT compared to CM (Supplementary Table 5A). We quantified SM, Cer, and dhCer in plasma and found a significant decrease in the proportion of Cer in CM (3.5 ± 1.2, mean ± SEM,%, 95% CI = 2.8–4.1) than CT (7.4 ± 4.5, mean ± SEM,%, 95% CI = 4.2–10.6). The AUC for Cer is 0.85 ± 0.09, 95% CI = 0.67–1.0, *p* = 0.0039, Figure 6A). Analyses of the two main clusters (#1 and #2, Figure 6B) show that plasma GPs and SPs are not good classifiers of CT and CM (Fisher’s exact test *p* > 0.9999).

### CSF GPs and SPs

We observed heterogeneity in GP levels in CSF for CT compared to CM patients. In general, PC and LPC levels were similar in CM than in CT (Supplementary Table 5B). However, the proportions of PAF compared to all lipids were higher in CT (0.3 ± 0.2, mean ± SEM%, 95% CI = 0.2–0.5) than CM (0.15 ± 0.023, mean ± SEM%, 95% CI = 0.1–0.2). The AUC for PAF in CSF is 0.89 ± 0.06 (mean ± SEM,%, 95% CI = 0.75–1.0, *p* = 0.0013), (Figure 6C). The SP levels did not attain statistical significance when calculated as ng/mL or expressed as a percent of GP/SP levels in CSF (Supplementary Table 5B). Cer levels were also higher in CM, but dhCer levels were similar for both groups, resulting in lower dhCer in CM (Supplementary Table 5B). Cluster analyses (Figure 6D) show that CSF GPs and SPs are good CT and CM identifiers (Fisher’s exact test *p* = 0.0154).

### CSF to Plasma GP/SP Ratios

To determine if there is a difference in GP and SP metabolism in CSF compared with plasma, we calculated the ratio of the proportion of lipid classes. The PAF CSF/plasma ratio was significantly lower in CM (0.3 ± 0.2, 95% CI = 0.2–0.5) than in CT (0.7 ± 0.2, 95% CI = 0.5–0.8) and the Cer ratio is significantly higher in CM (1.8 ± 0.8, 95% CI = 1.3–2.3) than CT (1.0 ± 0.4, 95% CI = 0.7–1.2). The AUC for PAF and Cer CSF/plasma ratios are 0.87 ± 0.07 (95% CI = 0.7–1.0, *p* = 0.0019), and 0.82 ± 0.09 (95% CI = 0.7–1.0, *p* = 0.0076), respectively (Figure 6E). The ratios of the other GP and SP classes were not different (Supplementary Table 5C). However, cluster analyses show that the ratios of GPs and SPs metabolism in CSF to plasma are excellent classifiers of CT and CM (Fisher’s exact test *p* = 0.0002) (Figure 6F). These data show differences in PAF and Cer formation in CSF and plasma for CT versus CM but similarities in the proportion of the other lipid classes.

### Phospholipase A_2_ Activity in Plasma and CSF

To determine if there was an increase in GP hydrolysis in CM, we measured PLA_2_ activity in plasma and CSF from CT and CM participants. CSF calcium-dependent PLA_2_ activity did not significantly differ in CT (7.2 ± 0.5 RFU/min/μg protein) and CM (7.9 ± 2.3 RUF/min/μg protein, *p* = 0.6047) CSF. Similarly, plasma PLA_2_ activity did not differ in CT (1.3 ± 0.3 RFU/min/μg) and CM (1.2 ± 0.3 RFU/min/μg, *p* = 0.4863).

## Discussion

Our studies show abnormalities in lipid metabolism in CM compared to healthy control participants, yet the changes differed markedly between CSF and plasma. Specifically, there is an increase in all groups (SAFAs, MUFAs, and PUFAs) of plasma UFAs in CM relative to CT. In contrast, only unesterified palmitoleic acid (C16:1) levels increased in CSF. Similar to plasma UFAs, mainly esterified SAFAs increased in CM, one esterified MUFA decreased (C16:1T), while another increased (C24:1), and none of the esterified n-3 PUFA nor the n-6 PUFAs increased in plasma. In addition, the unesterified plasma AA/DGLA ratio decreased in plasma, while the esterified AA/DGLA ratio was lower in CSF. In CSF, the plasma esterified elongase ratio was lower in CM, but there was no change in the elongase index. We quantified the major GPs and SPs in plasma and CSF and found a significant decrease in plasma Cer and CSF PAF in CM compared to CT. Cluster analyses show that CSF lipid metabolism and CSF ratio to plasma lipid ratio are good CT and CM classifiers. Together, these data show differences in lipid metabolism in CT and CM and headache-specific differences in plasma and CSF lipid metabolism. We summarize the findings of our study and their implications in Table 2. The most important lipid changes are associated with energy homeostasis, pain and inflammation, and insulin resistance. These differences suggest an abnormality in CM lipid metabolism that may be explored for understanding migraine pathophysiology, identifying biomarkers, and discovering new treatment strategies.

**TABLE 2:** Summary of lipid changes in CM and possible implications.

**Lipids**	**CM changes**	**Known function with potential CM implications**
SAFAs	Plasma unesterified (C14:0, C16:0, C18:0, eSAFA, C15:0, C17:0, oSAFA) and esterified SAFA species (C14:0, C22:0, C24:0, C15:0, C17:0, oSAFA) are higher in CM (Figures 1, 2).	Unesterified fatty acids are used for energy, and their levels increase in response to catecholamines, glucagon, or corticosteroids (Zhang and Ma, 2017). Dietary SAFAs worsen pain (Sekar et al., 2020). Higher unesterified plasma fatty acids may also link with insulin resistance proposed to be a part of migraine pathophysiology (Rao and Pearce, 1971; Perciaccante and Perciaccante, 2008). Both CNS and peripheral nervous system myelination depend on *de novo* fatty acid synthesis.
MUFAs	Plasma unesterified MUFAs (C14:1, C16:1, C16:1T, C18:1, C20:1, C24:1, C17:1, C19:1, oMUFA) levels are higher in CM. Esterified C16:1T is decreased while esterified C24:1 is increased in CM (Figure 3). There is no significant change in CSF MUFAs (Supplementary Table 1).	Palmitoleic acid (C16:1) is a proposed lipokine that decreases inflammation and is involved in glucose homeostasis and insulin resistance (Nunes and Rafacho, 2017; de Souza et al., 2018). C18:1 is a neurotrophic factor (Velasco et al., 2003) that promotes brain development and neuronal growth (Polo-Hernandez et al., 2014). 20:1 and C24:1 are major components of sphingolipids involved in myelination. Vitamin B12 deficiency increases oMUFAs in GPs isolated from myelin (Ramsey et al., 1977). Higher oMUFAs in CM may suggest vitamin imbalance (Kishimoto et al., 1973).
PUFAs	Higher plasma levels of unesterified n-3 fatty acids (C18:3n-3, C20:3n-3, C22:5n-3, C22:6n-3, n-3 PUFA) and unesterified n-6 fatty acids (C18:2n-6, C18:3n-6, dihomo-γ-C20:3n-6, C22:4n-6) in CM. There is no change in esterified n-3 pr n-6 PUFAs (Figure 4). There is no significant change in CSF (Supplementary Table 2).	n-3 PUFAs are anti-inflammatory and immunomodulatory (Tu et al., 2020), pro-resolving of inflammation and pain (Ji et al., 2011; Serhan and Levy, 2018) Elevated n-6 play a role in chronic pain (Prego-Dominguez et al., 2016; Shapiro et al., 2016; Lin et al., 2020), energy control, and inflammation (Chen et al., 2016; Dragano et al., 2017; Artiach and Back, 2020)
D5D	Decreased unesterified plasma and esterified CSF (Figure 5)	Higher D5D is associated with stroke (Daneshmand et al., 2017), inflammation and insulin resistance (Baugh et al., 2015; Pickens et al., 2016). The decrease in C20:4n-6/dihomo-γ-C20:3n-6 ratio may indicate AA’s preferential use for prostanoid formation. Such a process will increase prostaglandin formation and therefore enhance pain signaling.
Elongase	Decreased in esterified plasma fraction but does not change in CSF of CM compared with CT	Elongase is a metabolic checkpoint in energy regulation in rodents (Matsuzaka, 2021). In addition, Elongase products prevent dry eye disease (Sassa et al., 2018).
Cer	Plasma Cer is lower in CM (Figure 6A and Supplementary Table 5)	Cer is essential in energy metabolism, metabolic syndrome, and body weight regulation (Yang et al., 2009). Ameliorates energy homeostasis, lipid profile, and antioxidant systems (Zanieri et al., 2020). The Cer to the sphingosine-1-phosphate pathway is essential in pain (Salvemini et al., 2013).
PAF	As a proportion of all glycerophospholipids and sphingolipids, PAF is decreased in CSF (Figure 6B and Supplementary Table 5)	PAF is important in synaptic function, injury, and inflammation (Bazan and Allan, 1996), is a dual modulator involved in neuroprotection, plasticity and becomes neurotoxic when overproduced (Bazan, 1998; Tian and Bazan, 2005). PAF increases in migraine without aura at headache phase and then lower hours later (Sarchielli et al., 2004).

**FIGURE 7:**
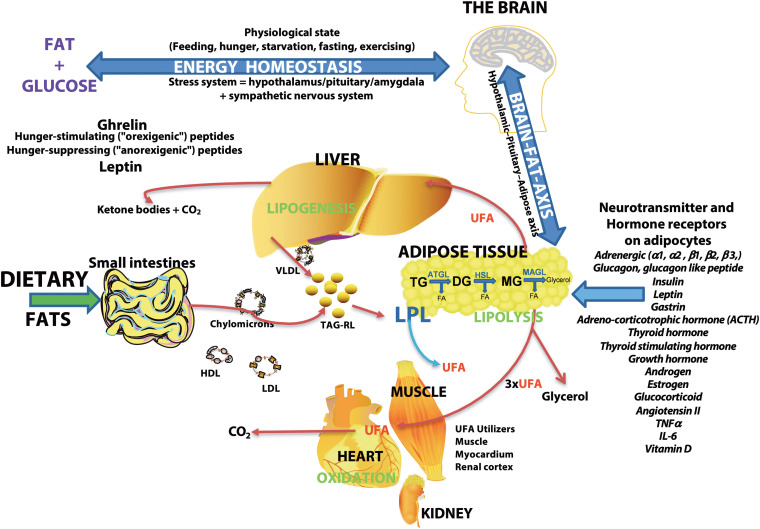
Schematic overview of the major sites of lipogenesis and lipolysis and their potential roles in generating unesterified fatty acids–The brain regulates the body’s physiologic state and is involved in energy homeostasis via hunger-stimulating (ghrelin) or hunger-suppressing (leptin) peptides. Dietary fats or fatty acids synthesized in cells are packaged into triacylglycerol-rich lipids (TAG-RL) or are associated with lipoproteins (VLDL, LDL, chylomicrons, and HDL) for transport to tissues. Extra TAG-RL is stored in adipose tissues. Studies show the expression of several neurotransmitters and hormone receptors in adipose tissue, suggesting an interaction of the brain and adipose tissue via the hypothalamic-pituitary-adipose axis or the Brain-Fat-Axis. In times of high energy demands or low blood glucose, sympathetic pathways may stimulate the release of fatty acids through receptor signaling pathways involving PKA/PKG, PKC, tyrosine kinase, and ERK1/ERK2. Unesterified fatty acids (UFA) are released from adipose tissues by lipoprotein lipase (LPL) or a combination of other lipases [adipose triglyceride lipase (ATGL), hormone-sensitive lipase (HSL), and monoacylglycerol lipase (MAGL)] that are regulated by neurotransmitters, hormones, and neuropeptides. The final lipolysis products are a glycerol molecule and three fatty acids. The released fatty acids are utilized by organs (heart, muscle, and renal cortex) for energy or modified in the liver (lipogenesis) or used to form ketone bodies. Lipolysis is stimulated by adrenergic signaling and glucagon and is inhibited by insulin. Thus, insulin resistance associated with migraines may result in the buildup of unesterified fatty acids in plasma.

As the major storage form of energy, lipid levels are controlled by the brain via hunger-stimulating or hunger-suppressing peptides (Figure 7). Fatty acids are the building blocks of the major lipid classes derived from the diet or synthesized by fatty acid synthetase. Fatty acids are then packaged into triacylglycerol-rich lipids (TAG-RL) or are bound to lipoproteins (VLDL, LDL, chylomicrons, and HDL) for transport to tissues. Excess triacylglycerol-rich lipids (TAG-RL) are stored in adipose tissues. Fatty acids in adipose tissue are released by catecholamine and hormonal stimulation of a combination of enzymes, including acyl-triglyceride lipase, HSL, and MAGL (Zechner et al., 2017). Recent studies show the expression of several neurotransmitters and hormone receptors on adipose tissue, suggesting an interaction of the brain and adipose tissue via the hypothalamic-pituitary-adipose axis or Brain-Fat-Axis (Pedersen et al., 1996; Bluher, 2013; Kim et al., 2015; Cignarelli et al., 2019; Starvaggi Cucuzza et al., 2020; Im et al., 2021). When glucose levels are low or when energy demand is high, sympathetic pathways may stimulate the release of fatty acids through receptor signaling pathways involving PKA/PKG, PKC, tyrosine kinase, and ERK1/ERK2 (Zechner et al., 2017). In addition to these cellular-derived fatty acids, extracellular fatty acids may be released by lipoprotein lipase (LPL) activity on lipoproteins (Coonrod et al., 1989; Coniglio, 1993; Sun et al., 2017). UFAs are used by organs such as the heart, muscle, renal cortex for energy or repackaged in the liver or used to form ketone bodies (Le Foll et al., 2014; Voros et al., 2018; Le Foll, 2019). Cellular lipolysis is stimulated by adrenergic signaling and glucagon and is inhibited by insulin (Zechner et al., 2017). Thus, the higher levels of plasma UFAs in CM may suggest an enhanced activation of lipolytic pathways. Since the balance of glucagon/catecholamines and insulin controls lipolysis, insulin resistance is suggested to be associated with migraine pathophysiology (Fava et al., 2014; Ozcan and Ozmen, 2019) may also account for the higher levels of UFAs in CM plasma.

In addition to energy regulation and insulin resistance, examining lipids that change in CM suggests abnormality in several enzyme and signaling pathways. Enzymes associated with SAFAs and MUFA metabolism include fatty acid synthase, elongases, and desaturases (Goodridge et al., 1986; Hiltunen et al., 2010). PUFAs are regulated by chain elongation, desaturation, and β-oxidation, while PAF levels are modulated by PLA_2_, acetyltransferase, and PAF acetylhydrolase (PAFA) (Sugiura et al., 1992).

### Saturated Fatty Acid Changes in CM

Total plasma UFAs and the levels of several unesterified SAFA and esterified SAFA species are higher in CM (Figures 1, 2). We summarize the implication of these changes in Table 2. The physiological implications include energy production (Lindsay, 1975; Jumpertz et al., 2012; Nakamura et al., 2014; Alcock and Lin, 2015; Cucchi et al., 2019), the trigger of the inflammatory response through via TLR4 signaling pathway associated with pain pathogenesis (Zhang and Ma, 2017), worsening pain (Sekar et al., 2020), and a link to insulin resistance that is proposed to be a part of migraine pathophysiology (Rao and Pearce, 1971; Perciaccante and Perciaccante, 2008; Bhoi et al., 2012; Ozcan and Ozmen, 2019). The levels and *de novo* synthesis of SAFAs are linked with CNS and peripheral nervous system myelination (Salles et al., 2002; Montani et al., 2018; Dimas et al., 2019), and fatty acid elongation is increased during active myelination in rodents (Morita et al., 2015). The changes in SAFA suggest a mirage of biochemical abnormalities in overall metabolism in CM participants.

### Monounsaturated Fatty Acid Changes in CM

Plasma levels of MUFAs were higher in CSF from CM subjects (Figure 3) while esterified C16:1T is decreased and esterified C24:1 is increased in CM (Figure 3). Palmitoleic acid (C16:1) is a proposed novel lipokine (Frigolet and Gutierrez-Aguilar, 2017) that decreases inflammation and is involved in glucose homeostasis and insulin resistance (Nunes and Rafacho, 2017; de Souza et al., 2018). C16:1 is derived from dietary sources or lipogenesis when palmitic acid formed by fatty acid synthase is converted to C16:1 by delta-9 desaturase or stearoyl-CoA desaturase (D9D or SCD1) activity. There are disagreements on the function of C16:1, but its roles on glucose homeostasis and insulin resistance (Nunes and Rafacho, 2017) are not disputed. Detrimental and beneficial effects of C16:1 have been reported in animal and human studies, likely related to dietary sources versus endogenous synthesis by the liver or adipose tissue (Mozaffarian et al.,2010a,b). In addition to insulin resistance and type-2 diabetes, C16:1 is implicated in mitochondrial permeability (Kurotani et al., 2012; Oyanagi et al., 2015), higher plasma levels are biomarkers of triglyceridemia and abdominal adiposity (Paillard et al., 2008), and increased risk of heart failure (Djousse et al., 2012). Higher C16:1 levels are also linked to carbohydrate intake and alcohol usage and are associated with metabolic risk factors. In contrast, consumption of purified C16:1 reduces C-reactive protein and improves metabolic risk factors (Bernstein et al., 2014).

In addition to palmitoleic acid, C18:1 (oleic acid), C20:1, and C24:1 are other MUFA species that increase in CM plasma. C18:1 is a neurotrophic factor (Velasco et al., 2003) that promotes brain development and neuronal growth (Polo-Hernandez et al., 2014), protects against oxidative stress (Guzman et al., 2016), and reversibly opens the blood–brain barrier (BBB) in rodents (Sztriha and Betz, 1991; Han et al., 2013). In addition, C20:1 and C24:1 are the primary components of sphingolipids involved in myelination. oMUFA are involved in vitamin B12 deficiency, resulting in an increase in odd-chain MUFAs in GPs isolated from myelin (Ramsey et al., 1977). Thus, higher oMUFAs in CM may suggest vitamin imbalance (Kishimoto et al., 1973).

Another MUFA that increased in CM is nervonic acid (C24:1). Nervonic acid is a very long chain (VLC) fatty acid and a significant brain myelin fraction component, accounting for 40% of total SM fatty acids (O’Brien and Rouser, 1964). C24:1 is formed from C18:1 by three successive elongation reactions, and lower C24:1 levels are reported in demyelinating diseases (Sargent et al., 1994). While the mitochondria oxidize most medium and long-chain fatty acids, peroxisomes are the oxidation site for VLC fatty acids (Sandhir et al., 1998). C24:1 levels are regulated by biosynthesis/dietary uptake and by β-oxidation, and the higher C24:1 in CM suggests altered VLC fatty acid metabolism in plasma. Plasma esterified elongase ratios were lower in CM, suggesting that the higher plasma C24:1 may be derived from cytosolic lipolysis. These data justify a further examination of these enzymes for a role in CM pathology.

### Polyunsaturated Fatty Acid Changes in CM

Another crucial fatty acid change in plasma is unesterified homo-γ-C20:3n-6 (DGLA). DGLA is formed by the elongation of GLA and is the intermediate in arachidonic acid (C20:4n-6, AA) formation. The D5D index (C20:4n-6/homo-γ-C20:3n-6) that measures the conversion of homo-γ-C20:3n-6 to C20:4n-6 is lower in CM compared to CT for unesterified plasma and esterified CSF. D5D is a rate-limiting enzyme in long-chain PUFA synthesis, and polymorphism has been reported in different races (Al-Hilal et al., 2013; Abdelmagid et al., 2015). The D5D decrease in plasma unesterified and CSF esterified D5D ratio has not been previously reported in CM. A higher level of homo-γ-C20:3n-6 is associated with insulin resistance, diabetes, obesity, contraceptive hormones usage, thyroid hormone status, and eating disorders (Araya et al., 2010; Swenne and Vessby, 2013; Pickens et al., 2016; Matsuda et al., 2017). DGLA also forms series-1 prostaglandins that are less potent than series-2 prostaglandins formed from arachidonic acid. PGE_1_ attenuates leukotriene B_4_ formation in human neutrophils (Chilton-Lopez et al., 1996; Kakutani et al., 2010), suggesting that higher DGLA in CM may have benefits. Our study justifies the design of intervention studies that alter the n-3 to n-6 ratios in headache and pain syndromes (Ramsden et al., 2010, 2011).

Given similarities in BMI for our CT and CM population (Table 1), it is likely that increased lipolysis (SAFAs, MUFAs, PUFAs) and enhanced synthesis (SAFAs) occurs in CM. Genome-wide association studies (GWAS) of subjects of European ancestry show that FADS1 and FADS2 polymorphism results in higher levels of MUFAs (C16:1, C18:1) and lower SAFA (C18:0) (Wu et al., 2013; Guan et al., 2014). These levels of MUFAs mirror the measurements we see in our study but not the changes in MUFA. These data suggest that different mechanisms, including genetic heritability and epigenetics, may alter our CM cohort’s fatty acid profiles.

### Cer Decreases in Plasma of CM

We found lower levels of Cer in CM plasma compared to controls (Figure 6A and Supplementary Table 5). Cer is important in energy metabolism, metabolic syndrome, body weight regulation (Yang et al., 2009) and ameliorates energy homeostasis, lipid profile, and antioxidant systems (Zanieri et al., 2020). Therefore, lower Cer levels may indicate an abnormality in these vital physiologic functions in CM participants.

### PAF Decrease in CSF of CM Patients

Platelet-activating factor metabolism involves PLA_2_ activity, acetyltransferase, and hydrolysis by PAFA. Both PC hydrolysis and lipid remodeling are known to regulate PAF formation (Chilton and Murphy, 1986). Lower remodeling in CM is consistent with bigger lipid pools and diminished capacity to form LPAF and PAF. Lower PAF levels may also be due to increased lipoprotein-associated phospholipase A_2_ (Lp-PLA_2_), an enzyme shown to increase in plasma of migraineurs implicated in increased cardiovascular risks and brain development (Clark, 2015). Future studies are necessary to examine the remodeling and modulation of lp-PLA_2_ pathways in CM compared with CT. Venous blood measurements have shown increased PAF levels during migraine without aura, and migraineurs have heightened sensitivity to PAF (Joseph et al., 1988; Sarchielli et al., 2004). However, LPC and PAF effects on the brain have been studied primarily on rodents. LPC is known to disrupt the BBB, will disturb brain lipid homeostasis, and PAF transiently increases BBB permeability and is a neuromodulator linked to brain injury (Yue and Feuerstein, 1994; Liu et al., 2001). PAF promotes neuroplasticity, so lower PAF levels in CM may point to habituation that mitigates the long-term cellular injury linked with these inflammatory mediators.

### Significance and Implication for Intervention Studies

Our studies show changes in lipid pathways and identify potential enzyme mechanisms accounting for these changes in CM. We show different plasma and CSF lipids changes, suggesting a manifestation of peripheral and central migraine pathology abnormalities in CM. We summarize the significant lipid changes and their likely significance in CM pathophysiology (Table 2). Our study’s major findings in lipid changes are in plasma lipolysis (SAFAs, MUFAs, PUFAs), lower Cer in plasma, and decreased CSF platelet-activating factor levels. An underlying outcome in the metabolic changes in CM is an imbalance with energy regulation, inflammatory pathways, and insulin resistance (Table 2). With a similar BMI for CT and CM subjects, metabolism and genetics rather than dietary differences likely account for these changes. These new findings can inform clinical care through dietary interventions and guide long-term pharmaceutical research. Since CM participants have abundant fatty acid levels, dietary intervention attempts at normalizing lipids should focus on D5D and elongase in CM rather than random dietary supplementation. For dietary, lifestyle modifications, or enzyme inhibitor studies, plasma UFA measures can be used to monitor efficiency. However, it should be noted that genetic differences and epigenetics may require personalized treatment strategies for people with metabolic diseases based on lipid profiles (Sergeant et al., 2016).

### Limitations

As with any human studies, diet, medication usage, and genetic heterogeneity are extremely difficult to control, and our study is underpowered by the number of plasma and CSF samples used to detect several metabolic changes. Participants did not fast before sample collection, and we did not obtain dietary history, and hormonal influence was not determined. More CM subjects are on NSAIDs, antioxidants and have more comorbid conditions, but most of these are not known to influence CSF lipid composition. As is with migraine prevalence that favors women, more women participated in our study (Table 1). Even with these limitations, CSF lipid differences between CM and CT that we identify underscore the central role of altered lipid metabolism in CM disorders.

## Conclusion

Higher plasma lipolysis involving neurotransmitter and hormonal signaling (Figure 7) may account for changes in lipid metabolism in CM. Hydrolysis and remodeling can alter lipids’ proportion and distribution and affect how bioactive lipids are formed, contributing to an altered metabolic state. Consequently, CM is considered a metabolic syndrome associated with dysfunctional lipid pathways that may influence energy homeostasis, pain pathways, and inflammatory signaling in the peripheral and central nervous systems. Future studies addressing individual enzyme polymorphisms and lipid classes and species will help unravel CM pathophysiology and identify personalized CM therapy.

## Data Availability Statement

The original contributions presented in the study are included in the article/Supplementary Material, further inquiries can be directed to the corresponding author/s.

## Ethics Statement

The studies involving human participants were reviewed and approved by IRB approval from Huntington Medical Research Institutes and Stanford University. The patients/participants provided their written informed consent to participate in this study.

## Author Contributions

AF, MH, and RC contributed to the conceptualization and study design. AF contributed to the writing of the original draft, manuscript preparation, validation, and supervision. MH and RC acquired funding, provided resources, and administered the clinical studies. KC prepared samples and acquired the data. KC and AF contributed to the formal data analysis. KC, YW, MH, RC, and AF contributed to the data curation. All authors contributed to the methodology, manuscript review and editing, and approval of the final manuscript.

## Conflict of Interest

The authors declare that the research was conducted in the absence of any commercial or financial relationships that could be construed as a potential conflict of interest.

## Publisher’s Note

All claims expressed in this article are solely those of the authors and do not necessarily represent those of their affiliated organizations, or those of the publisher, the editors and the reviewers. Any product that may be evaluated in this article, or claim that may be made by its manufacturer, is not guaranteed or endorsed by the publisher.

## Abbreviations

Cer: ceramide
dhCer: dihydroceramide
CM: chronic migraine
EFA: esterified fatty acids
GP: glycerophospholipid
LPAF: lyso platelet-activating factor
LPC: lysophosphatidylcholine
MUFA: monounsaturated fatty acid
PAF: platelet-activating factor
PC: phosphatidylcholine
SAFA: saturated fatty acid
SP: sphingolipids
UFA: unesterified fatty acids.
