# Supplementary material for: Plasma Lipolysis and Changes in Plasma and Cerebrospinal Fluid Signaling Lipids Reveal Abnormal Lipid Metabolism in Chronic Migraine
Source: Front Mol Neurosci. 2021 Aug 31;14:691733. doi: 10.3389/fnmol.2021.691733 (PMC8438335; doi:10.3389/fnmol.2021.691733)
Supplement: Supplementary file 1 [file Data_Sheet_1.pdf]

SUPPLEMENTARY DATA

Supplementary Table S1: Plasma unesterified fatty acids (ng/mL, Mean ± SEM (95% CI)

Fatty Acids	CT (n=10) unesterified saturated fatty acids (ng/mL, Mean ± SEM (95% CI) (ng/mL)	CM (n=15) unesterified saturated fatty acids (ng/mL, Mean ± SEM (95% CI) (ng/mL)	CM/CT*100
C14:0	2293.3 ± 262.3 (1699.9-2886.6)	4106.45 ± 430.5 (3183.1-5029.8)	179.1
C16:0	22957.4 ± 2023.6 (18379.7-27535.1)	37052.9 ± 2431.1 (31838.8-42267.0)	161.4
C18:0	17074.8 ± 2475.2 (11475.4-22674.2)	25447.5 ± 3091.3 (18817.3-32077.7)	149.0
C20:0	476.6 ± 111.7 (223.9-729.3)	1330.6 ± 374.4 (527.5-2133.6)	279.2
C22:0	282.6 ± 71.6 (120.6-444.5)	699.6 ± 169.1 (336.8-1062.4)	247.6
C24:0	447.7 ± 104.3 (211.9-683.5)	905.9 ± 196.6 (484.1-1327.6)	202.3
eSAFA	43532.3 ± 4420.4 (33532.7- 53532.0)	69542.9 ± 5978.4 (56720.4-82365.4)	159.7
C15:0	814.5 ± 92.8 (604.6-1024.3)	1306.9 ± 161.7 (960.1-1653.7)	160.5
C17:0	742.2 ± 114.4 (483.4-1001.0)	1417.1 ± 216.0 (953.8-1880.5)	190.9
C19:0	156.9 ± 41.8 (62.3-251.5)	470.8 ± 133.9 (183.7-757.9)	300.1
oSAFA	1713.5 ± 245.0 (1159.4-2267.7)	3194.8 ± 504.3 (2113.1-4276.4)	186.4
SAFA	45245.9 ± 4541.6 (34972.0-55519.8)	72737.7 ± 6424.9 (58957.6-86517.8)	160.8
	**CT unesterified monounsaturated fatty acids**	**CM unesterified monounsaturated fatty acids**	
C14:1n-5	158.9 ± 41.89 (64.2-253.5)	406.9 ± 68.5 (260.1-553.8)	256.2
C16:1n-7	758.5 ± 191.1 (326.2-1190.8)	2048.0± 375.2 (1243.3-2852.6)	270.0
C16:1Tn-9	4782.5 ± 535.0 (3572.2- 5992.8)	7623.3 ± 563.6 (6414.4-8832.1)	159.4
C18:1n-9	13562.1 ± 2732.1 (7381.5-19742.6)	29460.6 ±5027.8 (18677.1 40244.2)	217.2
C20:1n-9	473.5 ± 97.3 (253.5- 693.6)	1118.6 ± 156.6 (782.7-1454.5)	236.2
C22:1n-9	205.2 ± 55.9 (78.8- 331.6)	534.4 ± 133.7 (247.6-821.1)	260.5
C24:1n-9	114.0 ± 19.0 (71.1-157.0)	261.2 ± 48.2 (157.9-364.5)	229.1
eMUFA	20388.3 ± 3331.6 (12851.7-27924.8)	42097.7 ± 5709.3 (29852.4-54342.9)	206.5
C15:1n-5	53.6 ± 20.1 (8.1-99.1)	180.5 ± 50.31 (72.5-288.4)	336.5
C17:1n-7	218.0 ± 46.3 (113.2-322.8)	496.1 ± 69.3 (347.5-644.7)	227.6
C19:1n-9	76.1 ± 24.6 (20.4-131.8)	244.9 ± 68.7 (97.4- 392.3)	321.8
oMUFA	347.7 ± 89.5 (145.2-550.2)	921.4 ± 179.4 (536.6-1306.3)	265.0
MUFA	20735.9 ± 3364.6 (13124.6-28347.3)	43019.1 ± 5719.0 (30753.0-55285.2)	207.5
	**CT unesterified n-3 polyunsaturated fatty acids**	**CM unesterified n-3polyunsaturated fatty acids**	
C18:3n-3#	454.2 ± 111.6 (201.7-706.6)	905.9 ± 142.2 (601.0- 1210.9)	199.5
C20:3n-3	100.4 ± 14.0 (68.8-132.0)	185.7 ± 23.1 (136.1-235.2)	185.0
C20:5n-3	280.2 ± 52.1 (162.3-398.1)	646.3 ± 156.5 (310.7-981.9)	230.7
C22:3n-3	78.6 ± 21.3 (30.4-126.9)	163.8 ± 31.5 (96.2-231.4)	208.3
C22:5n-3	101.8 ± 15.9 (65.8-137.9)	219.8 ± 37.0 (140.4-299.2)	215.8
C22:6n-3	432.0 ± 72.8 (267.2-596.7)	768.2 ± 135.4 (477.7-1058.7)	177.8
N-3 PUFA	1447.1 ± 245.9 (890.9-2003.4)	2889.7 ± 503.7 (1809.3-3970.0)	199.7

	CT unesterified n-6 polyunsaturated fatty acids	CM unesterified n-6 polyunsaturated fatty acids	
**C18:2n-6**	11608.1 ± 2148.5 (6747.9-16468.2)	21022.9 ± 3028.4 (14527.7-7518.1)	181.1
**C18:3n-6#**	2425.2 ± 101.6 (2121.3-2813.0)	2752.4 ± 130.8 (2306.4-3070.6)	168.1
**C20:4n-6**	769.5 ± 103.7 (535.0- 1004.1)	1025.3 ± 96.2 (818.9-1231.6)	133.2
**homo-γ-C20:3n-6**	176.2 ± 23.8 (122.4-230.0)	328.6 ± 42.2 (238.2-419.1)	186.5
**C20:2n-6**	209.7± 25.6 (151.8-267.6)	393.6 ± 37.2 (313.7-473.4)	187.7
**C22:2n-6**	36.5 ± 10.6 (12.6-60.5)	102.3 ± 25.5 (47.6-157.0)	280.0
**C22:4n-6**	100.4 ± 10.5 (76.7-124.1)	158.4 ± 19.9 (115.8-201.1)	157.9
**N-6 PUFA**	52035.6 ± 10377.8 (28559.2-75511.9)	88798.2 ± 13243.1 (60394.7-117201.7)	170.6
**T-PUFA**	53482.6 ± 10453.8 (29834.5-77130.8)	91687.9 ± 13364.1 (63024.8-120351.0	171.4
**TOTAL**	252541.6 ± 28672.2 (187680.6-317402.5)	441203.4 ± 39683.8 (356090.1-526316.7)	174.7

eSAFA, even chain saturated fatty acid; oSAFA, odd chain saturated fatty acid; SAFA, the sum of eSAFA and oSAFA; eMUFA, even chain monounsaturated fatty acid; oMUFA, odd chain monounsaturated fatty acid; MUFA, the sum of eMUFA and oMUFA. #We found very high levels of C18:3n-6 in our samples due to the wrong calibration curve. Therefore, we used the standard curve for C18:3n-3 to estimate the levels C18:3n-6 since we noticed that both fatty acids have similar response ratio.

Supplementary Table S2: Plasma esterified fatty acids (Mean ± SEM (95% CI) (µg/mL)

Fatty Acids	CT (n=10) Esterified saturated fatty acids Mean ± SEM (95% CI) (µg/mL)	CM (n=15) Esterified saturated fatty acids Mean ± SEM (95% CI) (µg/mL)	CM/CT*100
**C14:0**	189.0 ± 6.9 (173.4-204.6)	312.7 ± 47.1 (211.6-413.8)	165.46
**C16:0**	2469.6 ± 117.2 (2204.6- 2734.6)	2981.4 ± 294.8 (2349.0-3613.7)	120.72
**C18:0**	1456.2 ±104.7 (1219.4-1693.0)	1491.5 ± 94.8 (1288.2- 1694.8)	102.42
**C20:0**	44.9 ± 3.8 (36.2- 53.6)	64.1 ± 6.1 (51.0-77.1)	142.77
**C22:0**	21.6 ± 4.5 (11.3-31.9)	37.8 ± 6.6 (23.6 + 52.0)	175.16
**C24:0**	19.2 ± 4.7 (8.7-29.8)	38.3 ± 7.4 (22.5- 54.1)	199.16
**eSAFA**	4200.4 ± 220.6 (3701.4-4699.5)	4925.7 ± 424.8 (4014.5-5836.9)	117.27
**C15:0**	61.9 ± 2.0 (57.4-66.5)	90.9 ± 10.7 (67.8-114.0)	146.78
**C17:0**	48.9 ± 1.8 (44.7- 53.2)	116.1 ± 34.3 (42.5-189.8)	237.44
**C19:0**	7.6 ± 0.5 (6.4- 8.8)	10.8 ± 1.7 (7.3-14.4)	143.18
**oSAFA**	118.4 ± 3.9 (109.5-127.3)	217.9 ± 45.8 (119.8-316.0)	184.00
**SAFA**	4318.9 ± 223.6 (3813.0- 4824.7)	5143.6 ± 464.3 (4147.8-6139.4)	119.10
	**CT esterified monounsaturated fatty acids**	**CM esterified monounsaturated fatty acids**	
**C14:1n-5**	2.3 ± 0.9 (1.4-3.1)	3.7± 0.8 (2.0- 5.4)	162.72
**C16:1n-7**	25.6 ± 3.1 (18.7-32.6)	57.7 ±11.5 (32.9-82.4)	225.20
**C16:1Tn-7**	279.3 ± 16.7 (241.6-317.0)1	195.1 ±18.8 (154.8-235.4)	69.86
**C18:1n-9**	500.9 ± 37.5 (416.2- 585.5)	519.3 ± 48.4 (415.4-623.2)	103.68
**C20:1n-9**	5.5 ± 0.7 (3.8-7.1)	10.6 ± 4.0 (2.0-19.1)	193.61
**C22:1n-9**	17.1 ± 2.2 (12.1- 22.1)	24.7 ±2.3 (19.7-29.6)	144.18
**C24:1n-9**	5.8 ± 0.6 (4.4-7.2)	13.7 ±1.4 (10.6-16.7)	235.84
**eMUFA**	849.9 ± 50.0 (734.8- 965.0)	849.3 ±65.8 (708.2-990.4)	99.93
**C15:1n-5**	0.4 ± 0.01 (0.3- 0.5)	0.3 ± 0.1 (0.2-0.5)	89.51
**C17:1n-7**	11.3 ± 0.8 (9.6-13.0)	17.2 ± 2.3 (12.2-22.1)	152.03
**C19:1n-9**	1.8 ± 0.1 (1.5-2.1)	1.9 ± 0.2 (1.5-2.4)	106.90
**oMUFA**	13.5 ± 0.8 (11.6-15.3)	19.4 ± 2.5 (14.1-24.8)	144.17
**MUFA**	863.3 ± 51.5 (747.- 979.7	868.7 ± 67.5 (724.0-1013.5)	100.62
	**CT esterified n-3 polyunsaturated fatty acids**	**CM esterified n-3 polyunsaturated fatty acids**	
**C18:3n-3**	9.4 ± 1.3 (6.4-12.3)	27.9 ± 11.4 (3.4-52.4)	297.73
**C18:4n-3**	1.0 ± 0.3 (0.3-1.8)	0.9 ± 0.2 (0.5-1.3	86.24
**C20:3n-3**	2.7 ± 0.4 (1.7-3.8)	3.2 ± 0.4 (2.4-4.1)	117.54
**C20:5n-3**	26.4 ± 2.6 (20.5-32.3)	27.5 ± 5.9 (15.0- 40.0)	104.07
**C22:3n-3**	0.6 ± 0.1 (0.3-0.8)	0.9 ± 0.2 (0.5-1.3)	165.40
**C22:5n-3**	6.4 ± 0.7 (4.9-7.9)	7.3 ± 0.5 (6.2-8.4)	114.21
**C22:6n-3**	32.3 ± 4.8 (21.4-43.2)	29.2 ± 2.2 (24.6-33.9)	90.47
**N-3 PUFA**	78.8 ± 8.1 (60.5-97.2)	97.0 ± 17.5 (59.5-134.5)	123.02
	**CT esterified n-6 polyunsaturated fatty acids**	**CM esterified n-6 polyunsaturated fatty acids**	
**C18:2n-6**	679.3 ± 47.6 (571.8-787.0	651.5 ± 30.1 (587.1-716.0)	95.91

**C18:3n-6#**	82.5 ± 9.4 (52.7-110.8)	87.8 ± 6.7 (73.4-102.3)	106.4
**C20:2n-6**	2.9 ± 0.4 (2.4-3.7)	5.4 ± 2.6 (2.6-8.3)	186.2
**C20:4n-6**	135.4 ± 17.5 (95.8-175.0)	117.2 ± 11.9 (91.7-142.7)	86.6
**homo-γ-C20:3n-6**	24.3 ± 2.4 (18.9-29.8)	34.7 ± 3.6 (26.5-42.9)	141.6
**C22:2n-6**	0.6 ± 0.1 (0.4-0.8)	0.9 ± 0.1 (0.7-1.1)	135.42
**C22:4n-6**	7.6 ± 0.7 (6.2-9.3)	7.6 ± 0.6 (6.4-8.8)	98.08
**N-6 PUFA**	1240.1 ± 96.2 (1022.4-1457.8)	1310.0 ± 27.4 (1036.8-1583.1)	105.63
**T-PUFA**	1318.9 ± 103.1 (1085.7-1552.1)	1406.9 ± 143.7 (1098.8-1715.0	106.67
**TOTAL**	6501.1 ± 271.0 (5888.1- 7114.1)	7419.3 ±628.9 (6070.5-8768.0)	114.12

eSAFA, even chain saturated fatty acid; oSAFA, odd chain saturated fatty acid; SAFA, the sum of eSAFA and oSAFA; eMUFA, even chain monounsaturated fatty acid; oMUFA, odd chain monounsaturated fatty acid; MUFA, the sum of eMUFA and oMUFA. #We used the standard curve for C18:3n-3 to estimate the levels C18:3n-6 as described on Table S1.

Supplementary Table S3: CSF unesterified fatty acids (Mean ± SEM (95% CI) (ng/mL)

Fatty Acids	CT (n=10) unesterified saturated fatty acids Mean ± SEM (95% CI) (ng/mL)	CM (n=15) unesterified saturated fatty acids Mean ± SEM (95% CI) (ng/mL)	CM/CT*100
C14:0	22.2 ± 1.2 (19.6-24.9)	21.6 ± 1.1 (19.-24.0)	97.212
C16:0	220.3 ±15.8 (184.6-256)	250.8 ± 28.6 (189.5-312)	113.84
C18:0	129.9 ± 4.5 (119.8 -140.1)	141.8 ± 10.5 (119.3-164.4)	109.16
C20:0	2.1 ± 0.1 (1.8-2.4)	2.2 ± 0.1 (2.0- 2.5)	108.04
C22:0	1.4 ± 0.1 (1.1-1.6)	1.3 ± 0.1 (1.2-1.4)	97.16
C24:0	2.2 ± 0.1 (1.9-2.5)	2.2 ± 0.2 (1.9-2.6)	100.81
eSAFA	378.1 ± 16.3 (341.1-415)	420 ± 38.4 (337.6-502.3)	111.08
C15:0	7.0 ± 0.3 (6.36-7.6)	7.0 ± 0.3 (6.4-7.5)	99.785
C17:0	4.1 ± 0.1 (3.8- 4.4)	4.1 ± 0.2 (3.8-4.4)	100.15
C19:0	0.5 ± 0.02 (0.5-0.6)	0.6 ± 0.03 (0.5-0.6)	108.91
oSAFA	11.6 ± 0.4 (10.7-12.5)	11.6 ± 0.4 (10.8-12.5)	100.35
SAFA	389.7 ± 16.6 (352.2 427.2)	431.6 ± 38.7 (348.6-514.6)	110.75
	**CT unesterified monounsaturated fatty acids**	**CT unesterified monounsaturated fatty acids**	
C14:1n-5	0.2 ± 0.04 (0.2-0.3)	0.3 ± 0.01 (0.2-0.4)	127.42
C16:1n-7	1.0 ± 0.1 (0.7-1.2)	1.8 ± 0.5 (0.7-3.0)	192.15
C16:1Tn-9	47.6 ± 3.7 (39.4-55.9)	57.5 ± 7.1 (42.2-72.8)	120.81
C18:1n-9	25.8 ± 5.4 (13.6-38.1)	29.5 ± 6.5 (15.8-43.1)	114.01
C20:1n-9	1.3 ± 0.1 (1.0-1.5)	1.4 ± 0.2 (1.1-1.7)	108.62
C22:1n-9	1.4 ± 0.1 (1.2-1.7)	1.5 ± 0.1 (1.3-1.7)	105.73
C24:1n-9	0.3 ± 0.03 (0.2-0.4)	0.4 ± 0.04 (0.3-0.4)	124.4
E-MUFA	79.6 ± 6.9 (64.0-95.2)	94.2 ± 13.5 (65.3-123.2)	118.44
C15:1n-5	0.04 ± 0.01 (0.03-0.06)	0.1 ± 0.01 (0.04-0.1	140.91
C17:1n-7	0.8 ± 0.1 (0.7-1.0)	0.8 ± 0.1 (0.7-1.1)	105.11
C19:1n-9	0.1 ± 0.01 (0.1-0.2)	0.2 ± 0.02 (0.1-0.2)	128.11
O-MUFA	1.0 ± 0.1 (0.8-1.2)	1.1 ± 0.1 (0.9-1.3)	109.5
T-MUFA	80.6 ± 6.9 (65.0-96.2)	95.3 ± 13.6 (66.2-124.5)	118.33
	**CT unesterified n-3 polyunsaturated fatty acids**	**CTunesterified n-3 polyunsaturated fatty acids**	
C18:3n-3	0.45 ± 0.04 (0.5-0.5)	0.64 ± 0.1 (0.4-0.9)	142.37
C20:3n-3	1.3 ± 0.1 (1- 1.6)	1.2 ± 0.1 (1.1-1.4)	92.578
C20:5n-3	4.8 ± 3.7 –(3.7-13.2)	1.3 ± 0.1 (1.0- 16)	26.756
C22:3n-3	N/D	0.2 ± 0.1 (-0.01 0.3)	
C22:5n-3	0.9 ± 0.6 (-0.5-2.4)	0.4 ± 0.1 (0.3-0.5)	41.41
C22:6n-3	8.1 ± 5.2 (-3.7-19.8)	3.7 ± 0.4 (2.8-4.6)	46.14
N-3 PUFA	15.5 ± 9.5 (-6.1-37.0)	7.4 ± 0.6 (6.1-8.7)	47.621
	**CT unesterified n-6 polyunsaturated fatty acids**	**CTunesterified n-6 polyunsaturated fatty acids**	
C18:2n-6	21.7 ± 15.5 (-13.3-56.7)	10.1 ± 2.8 (4.1-16.2)	46.725

**C18:3n-6#**	46.2 ± 2.1 (41.5-50.9)	44.51 ± 0.3 (43.9-45.1)	29.894
**C20:2n-6**	1.0 ± 0.1 (0.8-1.2)	1.1 ± 0.1 (0.9-1.4)	112.23
**homo-γ-C20:3n-6**	0.8 ± 0.03 (0.7-0.9)	0.9 ± 0.1 (0.8-1.1)	118.99
**C20:4n-6**	2.4 ± 0.3 (1.6-3.1)	2.8 ± 0.5 (1.9-3.8)	119.45
**C22:2n-6**	0.1 ± 0.01 (0.1-0.14)	0.2 ± 0.02 (0.1-0.2)	125.04
**C22:4n-6**	0.82 ± 0.1 (0.6-1.0)	0.8 ± 0.1 (0.7-0.9)	97.953
**N-6 PUFA**	115.5 ± 90.8 (-89.81-320.8)	42.5 ± 13.6 (13.5-71.6)	36.805
**T-PUFA**	130.9 ± 100.3 (-95.9 -357.8)	49.9 ± 14.0 (19.9-79.8)	38.105
**TOTAL**	1555 ± 139.4 (1240-1871)	1636 ±168.1 (1276-1997)	105.21

eSAFA, even chain saturated fatty acid; oSAFA, odd chain saturated fatty acid; SAFA, the sum of eSAFA and oSAFA; eMUFA, even chain monounsaturated fatty acid; oMUFA, odd chain monounsaturated fatty acid; MUFA, the sum of eMUFA and oMUFA. #We used the standard curve for C18:3n-3 to estimate the levels C18:3n-6 as described on Table S1.

Supplementary Table 4: CSF esterified fatty acids (Mean ± SEM (95% CI), ng/mL)

Fatty Acids	CT (n=10) saturated fatty acid Mean ± SEM (95% CI), ng/mL	CM (n=15) saturated fatty acid Mean ± SEM (95% CI)	CM/CT*100
C14:0	1130.0 3± 82.11 (944.3-1315.8)	2071.4 ± 960.6 (11.2-4131.5)	183.30
C16:0	18533.3 ± 1172.3 (15881.4-21185.2)	25614.6 ± 4157.3 (16698.4-34531.0)	138.21
C18:0	14008.393 ± 1112.6 (11491.6-16525.2)	18676.3 ± 3798.6 (10529.2-26823.4)	133.32
C20:0	343.3 ± 28.1 (279.8- 406.8)	333.1 ± 21.7 (286.7-379.6)	97.04
C22:0	287.9 ± 21.3 (239.7- 336.0)	360.9 ± 34.0 (287.9-433.9)	125.36
C24:0	339.0 ± 34.7 (260.4-417.5)	382.4 ± 32.7 (312.2-452.6)	112.83
eSAFA	34641.8 ± 2286.7 (29468.9-39814.8)	47438.6 ± 8801.0 (28562.3-66314.9)	136.94
C15:0	361.2 ± 22.9 (309.3-412.8)	496.0 ± 99.3 (283.0-709.0)	137.39
C17:0	265.6 ± 22.5 (214.8-316.4)	486.5 ±199.1 (59.6-913.5)	183.18
C19:0	55.8 ± 4.2 (46.3-65.3)	414.9 ± 358.5 (-354.05324-1183.9)	743.88
oSAFA	682.4 ± 48.0 (573.8-791.0)	1397.5 ± 654.5 (-6.3-2801.2)	204.79
SAFA	35324.2 ± 2329.3 (30055.0-40593.5)	48836.1 ± 9420.2 (28631.8-69040.4)	138.25
	**CT monounsaturated fatty acid Mean ± SEM (95% CI)**	**CM monounsaturated fatty acid Mean ± SEM (95% CI)**	
C14:1n-5	5.6 ± 0 .5 (4.4-6.8)	16.7 ± 9.5 (-3.7-37.1)	298.41
C16:1n-7	225.6 ± 22.7 (174.3- 276.9)	449.8 ± 92.0 (252.4-647.2)	199.38
C16:1Tn-7	6445.0 ± 404.3 (5530.4-7359.5)	9334.0 ± 1531.8 (6048.7-12619.3)	144.83
C18:1n-9	8646.2 ± 549.8 (7402.5-9890.0)	13283.3 ± 2217.9 (8526.5-18040.2)	153.63
C20:1n-9	261.8 ± 19.2 (218.3-305.3)	429.1 ± 94.6 (226.2-632.1)	163.90
C22:1n-9	2106.1 ± 250.4 (1539.6-2672.6)	2493.5 ± 251.9 (1953.2-3033.9)	118.39
C24:1n-9	699.4 ± 68.2 (545.0-853.7)	999.4 ± 107.9 (767.9-1230.9)	142.90
eMUFA	18029.3 ±1080.3 (15585.4-20473.2)	26388.9 ± 4015.7 (17776.1-35001.8)	146.37
C15:1n-5	1.0 ± 0.1 (0.8-1.3)	1.7 ± 0.4 (0.8-2.5)	164.20
C17:1n-7	72.7 ± 5.2 (61.0-84.3)	119.6 ±25.4 (65.2-173.9)	164.56
C19:1n-9	13.4 ± 0.9 (11.4- 15.4)	15.4 ± 1.4 (12.3-18.4)	114.33
oMUFA	87.1 ± 5.5 (74.6-99.6)	136.6 ± 26.7 (79.4-193.8)	156.82
MUFA	18116.4 ± 1084.9 (15662.1-20570.6)	26525.5 ± 4041.0 (17858.4-35192.6)	146.42
	**CT n-3 polyunsaturated fatty acid Mean ± SEM (95% CI)**	**CM n-3 polyunsaturated fatty acid Mean ± SEM (95% CI)**	
C18:3n-3	179.8 ± 24.8 (123.7- 235.8)	589.2 ± 249.3 (54.6-1123.9)	327.78
C20:3n-3	27.6 ± 2.0 (23.2- 32.0)	74.7 ± 40.1 (-11.4-160.8)	270.42
C20:5n-3	104.3 ± 9.1 (83.9- 124.8)	392.7 ± 251.2 (-146.1-931.5)	376.38
C22:3n-3	9.1 ± 0.7 (7.6- 10.7)	18.0 ± 6.6 (3.8- 32.2)	196.83
C22:5n-3	141.7 ± 7.9 (123.9-159.5)	181.8 ± 28.7 (120.3-243.3)	128.29
C22:6n-3	1689.8 ± 92.5 (1480.5- 1899.1)	1982.9 ± 210.5 (1531.4-2434.4)	117.35
N-3 PUFA	2152.3 ± 110.9 (1901.5-2403.1)	3239.3 ± 591.4 (1970.9-4507.7)	150.50
	**CT n-6 polyunsaturated fatty acid Mean ± SEM (95% CI)**	**CM n-6 polyunsaturated fatty acid Mean ± SEM (95% CI)**	
C18:2n-6	3382.0 ± 433.9 (2400.5-4363.5)	5952.1 ± 1264.8 (3239.4-8664.7)	175.99
C18:3n-6[#]	99.7 ± 1.4 (96.5-102.8)	124.6 ± 15.7 (90.9-158.3)	315.68

**C20:2n-6**	72.1 ± 6.1 (58.4-85.8)	144.0 ± 37.5 (63.6 -224.3)	199.66
**homo-γ-C20:3n-6**	292.6 ± 26.6 (232.4-352.8)	451.3 ± 48.8 (346.7- 555.9)	154.26
**C20:4n-6**	2565.7 ± 174.2 (2171.7-2959.7)	3414.5 ± 598.3 (2131.2 4697.8)	133.08
**C22:2n-6**	54.1 ± 5.0 (42.9-65.2)	74.2 ± 9.7 (53.3 - 95.1)	137.23
**C22:4n-6**	377.6 ± 27.6 (315.- 440.1)	413.6 ± 44.1 (319.1- 508.1)	109.54
**N-6 PUFA**	8860.0 ± 843.7 (6951.4-10768.7)	17129.4 ± 4039.2 (8466.3 - 25792.5)	193.33
**T-PUFA**	11012.3 ± 895.0 (8987.8-13036.9)	20368.7 ± 4597.2 (10508.6-30228.8)	184.96
**TOTAL**	173846.8 ± 10590.1 (149890.4-197803.2)	250309.7 ± 43854.1 (156252.1-344367.4)	143.98

eSAFA, even chain saturated fatty acid; oSAFA, odd chain saturated fatty acid; SAFA, the sum of eSAFA and oSAFA; eMUFA, even chain monounsaturated fatty acid; oMUFA, odd chain monounsaturated fatty acid; MUFA, the sum of eMUFA and oMUFA. [#]We used the standard curve for C18:3n-3 to estimate the levels C18:3n-6 as described on Table S1.

Supplementary Table 5: Glycerophospholipids (GPs) and sphingolipids (SPs) in CT and CM

Supplementary Table 5A – Plasma GPs and SPs (Mean ± SEM (95% CI), %)

Lipids	CT, n=10<br>(% Total GP & SPs)	CM, n=15<br>(% Total GP & SP)	p-value	q value
PC	60.7 ± 4.7 (57.3 – 64.0)	64.3 ± 3.2 (62.5 – 66.1)	0.0709	0.1910
LPC	1.5 ± 0.3(1.3-1.7)	1.6 ± 0.4(1.4-1.8)	0.8918	0.9443
LPAF	1.0 ± 0.3 (0.8 – 1.3)	0.9 ± 0.2(0.8- 1.2)	0.1963	0.3965
PAF	0.5 ± 01 (0.4 – 0.1)	0.5 ± 0.1(0.4-0.5)	0.3669	0.5929
SM	13.3 ± 1.6 (12.2 –14.4)	15.3 ± 1.6(14.4-16.1)	0.0137	0.0554
Cer	7.4 ± 4.5(4.2-0.6)	3.5 ± 1.2 (2.8-4.1)	0.0029	0.0235
dhCer	8.2 ± 3.4 (5.7 – 10.5)	6.5 ± 1.8 (5.5 – 7.5)	0.4952	0.6669

We obtained P values using the Multiple Mann-Whitney test that compare ranks with multiple comparisons adjustment for False Discovery Rate (FDR) using the Two-Stage step-up method of Benjamini, Krieger, Yekutieli. The adjusted p values (q) show a significant decrease in the proportion of plasma Cer in CM patients.

Supplementary Table 5B: CSF GPs and SMs (Mean ± SEM (95% CI), %)

Lipids	CT, n=10<br>(% Total GP & SPs)	CM, n=15<br>(% Total GP & SP)	p-value	q value
PC	73.0 ± 0.7 (71.4 – 74.6)	72.4 ± 1.3 (69.6 - 75.2)	0.8065	0.8146
LPC	0.06 ± 0.02 (0.02 – 0.1)	0.05 ± 0.02 (0.01 – 0.1)	0.2853	0.5042
LPAF	0.4 ± 0.1 (0.2 – 0.6)	0.2 ± 0.04(0.1-0.3)	0.0910	0.3216
PAF	0.34 ± 0.04 (0.3 – 0.4)	0.15 ± 0.03(0.1– 0.2)	0.0007	0.0049
SM	16.5 ± 0.6 (15.1 – 17.9)	17.5 ± 0.4(16.6 – 18.3)	0.2380	0.5042
Cer	1.4 ± 0.1(1.2 – 1.6)	1.7 ± 0.2 (1.2 -2.2)	0.9783	0.8645
dhCer	6.1 ± 0.7 (4.6 – 7.7)	4.9 ± 0.8 (3.3 – 6.5)	0.3970	0.5613

P values were obtained as described above in Table 2A. The adjusted p values (q) show a significant decrease in the CSF PAF fraction in CM patients. However, the proportions of PAF compared to all lipids were lower in CM (0.3 ± 0.2, mean ± SEM % , 95 % CI = 0.2-0.5) than CT (0.7 ± 0 .2, mean ± SEM %, 95 % CI = 0.5-0.8).

Supplementary Table 5C: CSF/Plasma GPs and SPs (Mean ± SEM (95% CI), Ratio)

Lipids	CT, n=10<br>Ratio of CSF/plasma	CM, n=15<br>Ratio of CSF/plasma	p-value	q value
PC	1.2 ± 0.1 (1.1 – 1.3)	1.1 ± 0.1 (1.1 – 1.2)	0.1775	0.2313
LPC	0.04 ±0.03 (0.01 –0.04)	0.04 ± 0.07 (0.0 – 0.08)	0.1963	0.2313
LPAF	0.4 ± 0.2 (0.2 – 0.6)	0.3 ± 0.2 (0.2-0.4)	0.1439	0.2313
PAF	0.7 ± 0.2 (0.5 – 0.8)	0.3 ± 0.2 (0.2 – 0.5)	0.0012	0.0082
SM	1.2 ± 0.2 (1.1 – 1.4)	1.2 ± 0.2 (0.2 – 0.5)	0.1600	0.2313
Cer	1.0 ± 0.4 (0.7 – 1.2)	1.8 ± 0.9 (1.3 -2.3)	0.0066	0.0233
dhCer	2.1 ± 1.3 (1.2 – 3.0)	2.2 ± 1.6 (1.3 – 3.0)	0.9350	0.8263

The proportion of GPs and SPs was calculated in CSF and plasma, and the ratio of CSF to plasma was obtained. P values were as described in Table 2A. The adjusted p values (q) show a significant decrease in CSF to plasma ratio of PAF and an increase in CSF to plasma ratio of Cer in CM patients.
